# Prolactin Monitoring for Inpatients on Antipsychotic Drugs: A Clinical Audit

**DOI:** 10.1192/bjo.2024.592

**Published:** 2024-08-01

**Authors:** Farida Kotait, Sheena Parmar, Nilamadhab Kar

**Affiliations:** 1 New Cross Hospital, Wolverhampton, United Kingdom; 2 Penn Hospital, Wolverhampton, United Kingdom

## Abstract

**Aims:**

Many antipsychotics are known to adversely affect prolactin levels causing hyperprolactinemia. National Institute for Health and Care Excellence (NICE) guidelines have suggested monitoring of prolactin levels. It specifies that prolactin should be checked 6 months after starting treatment, then every 12 months; and to ask about symptoms of raised prolactin which include low libido, sexual dysfunction, menstrual abnormalities, gynaecomastia, and galactorrhoea. This also mentions that it is not required for aripiprazole, clozapine, quetiapine, or olanzapine (less than 20 mg daily). We intended to audit the monitoring of prolactin in a sample of inpatients who are on antipsychotic drugs and to check whether action was taken in the event of a high prolactin level.

**Methods:**

All the adult inpatients of a general psychiatric hospital on a specific date (34, 16 (47.1%) female and 18 (52.9%) male patients), who were on antipsychotics were considered for the audit. We checked the antipsychotic drugs, prolactin assessment within one year and level, action taken if there was hyperprolactinemia. The data was collected from electronic patient records and medication charts.

**Results:**

The mean age of the sample was 39.1 ± 14.2 years (range 18–63). Most inpatients (91.2%, 31/34) were on antipsychotics and 25.8% (8/31) were on two antipsychotic drugs. Prolactin was measured in 80.6% (25/31) patients in the last year, with 48% (12/25) having hyperprolactinemia; and amongst these action was taken in 5 (41.7%). Hyperprolactinemia was present in 58.3% of female and 38.5% of male patients. Specifically, out of 31 patients, 14 (45.2%) were on antipsychotic drugs that need monitoring, and 9 (74.3%) of them had taken it for at least one year. Out of these 9 patients, prolactin was measured in 8 (88.9%) patients in the last year, it was elevated in 5 (55.6%), action was taken in 3 (60%) and action was not clear in 2 (40%) patients.

**Conclusion:**

Almost half of the patients on antipsychotic drugs had hyperprolactinemia, highlighting the need to monitor prolactin levels. Along with this, symptoms of hyperprolactinemia should be consistently checked in routine clinical evaluations. Clinician and patient education regarding hyperprolactinemia and its symptoms might improve its monitoring.

